# Photobiomodulation for Pain, Healing, and Analgesic Use after Perineal Lacerations: A Double-Blind Randomized Controlled Trial

**DOI:** 10.1007/s00192-026-06686-5

**Published:** 2026-06-17

**Authors:** Thais de Castro, Laíza Helena Viana, Simone Botelho, Lígia Sousa Marino

**Affiliations:** 1 Institute of Motor Sciences, Physiotherapy, Federal University of Alfenas, Jovino Fernandes Sales Avenue, Alfenas, 2600 Brazil; 2 University of Campinas–School of Medical Sciences–Postgraduate Program in Surgical Science (UNICAMP)–Campinas, São Paulo, Brazil

**Keywords:** Episiotomy, Low-level laser therapy, Photobiomodulation, Puerperium

## Abstract

**Introduction and Hypothesis:**

Approximately 60% of women experience some degree of perineal laceration following vaginal delivery. Although there is evidence supporting the use of photobiomodulation (PBM) for pain relief and wound healing in various conditions, there is a lack of studies on its effect on these lacerations. Our hypothesis is that PBM aids in pain management and healing in perineal lacerations/episiotomies in hospitalized women.

**Materials and Methods:**

A randomized, controlled, double-blind clinical trial in women with grade I/II lacerations and/or episiotomy, assigned to the intervention group (660 nm; 4 J) or placebo group (device turned off). Primary outcome was perineal pain at rest and during movements, measured using the numeric rating scale. Secondary outcome was healing, measured using the REEDA (redness, edema, ecchymosis, discharge, approximation) scale. Additionally, in-hospital analgesic consumption, self-perception of perineal laceration healing, and satisfaction with the intervention were analyzed. Analysis performed pre-intervention (6–12 h postpartum), post-intervention 1 (6–12 h post-first intervention), post-intervention 2 (12–18 h post-first intervention), and follow-up (7–15 days postpartum). Analysis was performed on an intention-to-treat basis using the Mann–Whitney test.

**Results:**

A total of 59 women participated (intervention 30; placebo 29), most of whom were primiparous. The intragroup analysis shows a reduction in perineal pain during rest and movements, with improved healing in both groups. No difference was observed in intergroup analysis. The intervention group consumed fewer analgesics and had a longer interval between doses than the placebo group.

**Conclusion:**

Photobiomodulation (660 nm; 4 J) applied within 24 h postpartum, did not result in clinical improvement in pain or healing in women with perineal lacerations/episiotomies.

## Introduction

Perineal lacerations affect approximately 60% of women who give birth vaginally [1] and are classified into four degrees based on their severity. The extent of the injury and the associated symptoms vary according to the degree of the laceration [2–4].

Spontaneous first- and second-degree lacerations, as well as episiotomy, are the most common forms of perineal laceration in vaginal delivery [1]. Despite their lesser severity, they can cause pain and discomfort [4]. Third- and fourth-degree lacerations, known as OASIs (obstetric anal sphincter injuries), are lacerations that involve the anal sphincter and affect 2% to 3% of women [1]. These lacerations are associated with greater physical and psychological impairment and constitute a significant risk factor for the development of anal incontinence [3]. Perineal pain is present in about 60% of women in the first few hours after delivery and may persist long term, especially in those who experienced a laceration [5]. Any degree of perineal laceration can cause pain and functional impairment, making it difficult to care for the newborn and to return to daily activities in the postpartum period [3, 4].

The healing process begins immediately after the perineal laceration, particularly through platelet activation and immune system action, triggering an inflammatory process [6]. The management of inflammatory signs, particularly pain, edema, and hyperemia, is one of the primary clinical concerns immediately following vaginal delivery, as these signs can compromise a woman’s overall well-being [7, 8].

Assessing the healing of perineal lacerations using quantitative methods can be challenging [8]. Studies predominantly use the redness, edema, ecchymosis, discharge, approximation (REEDA) scale for the clinical assessment of inflammatory signs and tissue repair, including the items hyperemia, edema, ecchymosis, discharge, and edge approximation. This scale can be used for any type of perineal laceration, starting in the first hour postpartum. [8, 9].

Perineal lacerations are usually managed with pharmacological interventions, such as analgesics and nonsteroidal anti-inflammatory drugs, and with nonpharmacological interventions, such as cryotherapy [7]. In some cases, surgical repair is necessary [7]. Photobiomodulation therapy (PBM) is well established in the literature as a nonpharmacological strategy that uses non-ionizing light to promote analgesic and healing effects in various types of injuries [10, 11]. PBM stimulates cell proliferation by increasing ATP production and cellular metabolism. It also improves microcirculation by inducing the release of endothelial growth factors, promoting angiogenesis, and modulating the inflammatory response caused by the injury, thereby contributing to pain relief and healing [10, 12].

However, there is no consensus in the literature regarding the benefits of PBM for perineal lacerations [13]. Studies on the topic have limited methodological quality and lack consensus regarding the protocols used, and to date, they have been scarce [13]. In addition to the mentioned effects, PBM may have the potential to reduce the use of analgesics owing to its effects on the modulation of inflammatory mediators, such as oxytocins and prostaglandins [10].

Given the prevalence and need for research on conservative interventions for perineal lacerations, this study evaluated an in-hospital protocol for the application of PBM (660 nm; 4 J) on pain and healing of first- and second-degree perineal lacerations and/or episiotomies in women after vaginal delivery. Additionally, the use of analgesics, self-perception of perineal laceration healing and the satisfaction of the women with the proposed intervention were analyzed.

## Materials and Methods

### Study Design and Ethical Aspects

This is a randomized, controlled, double-blind, parallel-group clinical trial, approved by the Research Ethics Committee of Federal University of Alfenas/MG, under opinion no. 6.463.428. The study was registered with the Brazilian Clinical Trials Registry (ReBEC), under No. RBR-3t8pw38. All the women signed the Informed Consent Form. All the study procedures comply with the Consolidated Standards of Reporting Trials guidelines, 2025. The women did not participate in the planning, conduct, or dissemination of this study.

### Location

The research was conducted at a maternity hospital located in the municipality of Alfenas, Minas Gerais, Brazil, following formal authorization from the institution.

### Population and Eligibility Criteria

The study was conducted among women who had given birth vaginally at the aforementioned maternity ward and who expressed interest in participating. The inclusion criteria were defined as: age over 18 years; primiparous or multiparous women; women who had undergone vaginal delivery with first- or second-degree perineal lacerations; women who had undergone vaginal delivery with episiotomy; approximation of the edges of the laceration by suture (primary closure); pain rated at least 1 point on the numerical pain scale; and ability to understand simple commands after verbal instruction. Exclusion criteria included: refusal to participate in the protocol; presence of systemic or local infections at the site of the injury; twin pregnancy; hemodynamic instability; and postpartum complications (bleeding).

### Intervention and Comparator

The women in the intervention group received PBM, whereas those in the placebo group received a placebo, immediately following the assessments in the maternity ward. For PBM application, the DUO MMO® laser device, São Carlos, São Paulo, Brazil (ANVISA Registration: 80,051,420,022), was used, with an output power of 100 mW, irradiance of 3.33 mW/cm^2^, and a spot size of 0.03 cm^2^. A 660-nm wavelength pen (Gallium-Aluminum Arsenide [GaAlAs]) with a red (visible) electromagnetic spectrum was used. The mode was continuous, with a dose of 133.33 J/cm^2^, energy of 4 J, and an application time of 40 s per point, as determined by the device itself. A trained and qualified physical therapist applied PBM at specific points, maintaining a distance of 0.5 cm between the device’s probe and the woman’s skin, positioned perpendicularly [14]. The tip of the device’s probe was sanitized between the irradiation sessions with 70% alcohol and covered with a flexible plastic material (polyvinyl chloride) [14].

**Fig. 1:**
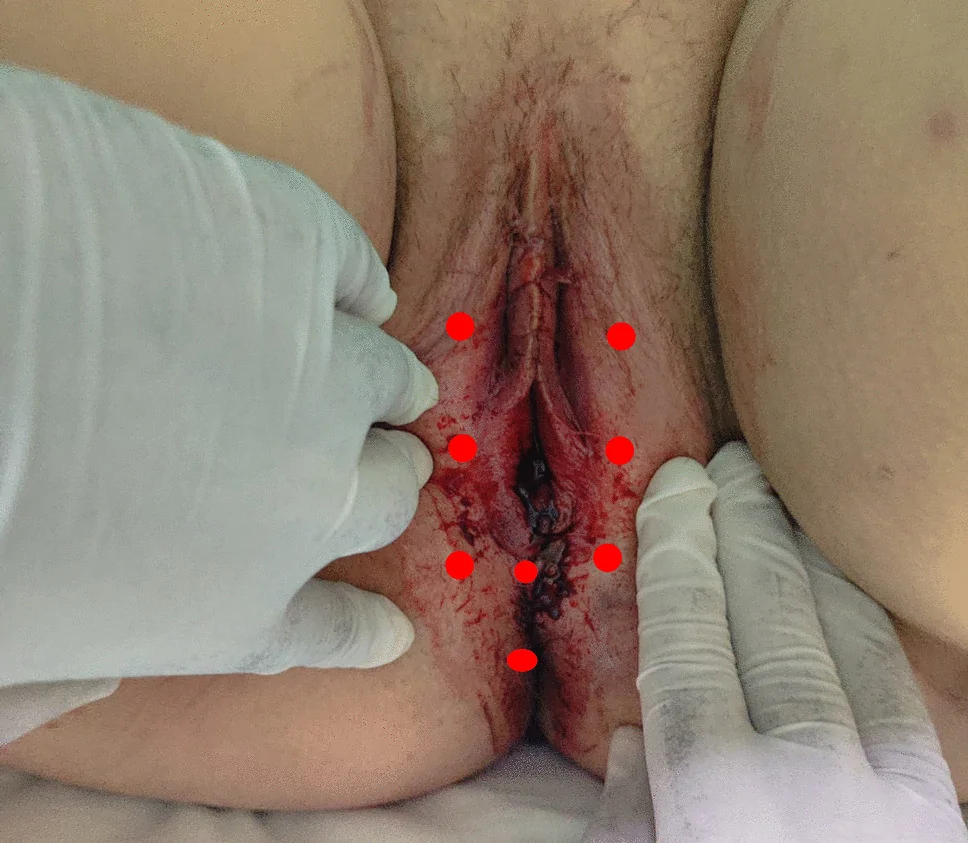
Example of photobiomodulation application points. Source: Prepared by the author

Both the physical therapist and the woman wore protective eyewear for added safety. For the procedure, the woman was positioned in the supine position, with her hips and knees flexed and her feet resting on the examination table; a hospital sheet was placed over her to minimize the embarrassment. The number of PBM points applied depended on the extent of the laceration, with a distance of 1 cm between points. In addition, three points were applied on each side along the outer edges of the labia majora (Fig. 1). For the placebo group, the same points were marked, and the device was positioned as if it were in operation, but without a programmed dosage.

### Sample Characterization

The data used to characterize the sample were extracted from medical records by the evaluating researcher during the pre-intervention assessment. The instrument included sociodemographic data (name, date of birth, and age), obstetric information (gestational age, number of previous deliveries, history of abortion, duration of the expulsion stage, fetal presentation, and newborn weight), as well as the presence and degree of spontaneous perineal lacerations and the performance of episiotomy.

### Outcomes

The women were assessed at four time points for primary outcomes and at three time points for secondary and additional outcomes: pre-intervention (between 6 and 12 h after delivery); post-intervention 1 (between 6 and 12 h after the first intervention); post-intervention 2 (between 12 and 18 h after the first intervention; in-hospital); and between 7 and 15 days after the delivery, via text messages. The intervention or placebo was administered immediately after the pre-intervention assessment and re-administered after post-intervention 1, totaling two interventions performed in the maternity ward.

#### Primary Outcome

Pain was assessed using the Numeric Rating Scale (NRS), a tool that measures pain on a scale from 0 to 10, where 0 corresponds to no pain and 10 to the worst pain imaginable. The women reported the pain score for perineal pain at rest, during functional movements [15, 16]. After the hospital discharge, the researcher responsible for the assessment contacted the women via text messages to evaluate perineal pain at rest using the NRS scale [17]. Scores are presented as mean and standard deviation.

#### Secondary Outcome

Healing was assessed using the REEDA scale. The REEDA scale assesses the healing of perineal lacerations, including spontaneous lacerations and episiotomies [8, 9]. Each item ranges from 0 to 3 points, with a maximum score of 15; higher scores indicate a worse local condition. To measure distances, a disposable measuring tape graduated from 0 to 6 cm, with markings in millimeters, was used.

#### Additional Outcome

In accordance with the institutional protocol for the management of perineal lacerations, a nonsteroidal anti-inflammatory drug (NSAID) and an opioid analgesic were routinely prescribed. A simple analgesic (non-anti-inflammatory and non-opioid) was administered as needed, upon the woman’s request or at the physician’s discretion. Data on medication consumption were obtained from each woman’s medical records, considering whether the medication was prescribed and the interval between doses.

After hospital discharge, the researcher responsible for the assessment contacted the women via text messages to evaluate their self-perception of perineal laceration healing (1: improved a lot; 2: improved a little; 3: no change; 4: worsened a little; 5: worsened a lot) and their satisfaction with the intervention (1: very satisfied; 2: satisfied; 3: neither satisfied nor dissatisfied; 4: dissatisfied; 5: very dissatisfied) [17]. Scores are presented as mean and standard deviation for quantitative analysis and as percentages for qualitative analysis.

### Sample Size

A pilot study was conducted with 12 women (6 in the intervention group and 6 in the placebo group). The sample size was calculated based on the primary outcome (perineal pain at rest), using G*Power 3.1.9.2 software and a t test, with a statistical power (1–β) of 0.95, a Type I error of 0.05, and an effect size of 1.02. The estimated sample size was 52 women (26 per group). The pilot study was incorporated into the main study, maintaining the same experimental protocol.

### Randomization and Blinding

Randomization was performed in blocks at a 1:1 ratio by an independent researcher (LSM), not involved in recruitment, using the Research Randomizer website, ensuring random and blinded allocation. The women were assigned to the intervention group or the placebo group according to the generated sequence. The blocks were sent directly to the researcher responsible for the intervention (TC). The researcher responsible for the evaluation was unaware of each participant’s group (LHV). The use of the placebo group ensured the blinding of the women and the evaluating researcher, characterizing the study as double-blind.

### Statistical Analysis

The data were tabulated in Microsoft Excel® 2021. Quantitative variables were described using the mean and standard deviation. Normality was assessed using the Shapiro–Wilk test. For normally distributed data, the *t* test for independent samples was used for intergroups analysis, and repeated-measures analysis of variance with a Bonferroni post hoc test was used for intragroup analysis. Non-normal variables were analyzed using the Mann–Whitney (between-group) and Friedman (within-group) tests.

In the intergroup analysis of qualitative variables, the Chi-squared test was used, with a description by frequency and percentage. For the intragroup analyses, Cochran’s Q test was used, with multiple comparisons via post hoc tests.

Medication consumption was analyzed as a dichotomous variable (1: yes; 2: no), using the Chi-squared test for the intergroup analysis. The interval between analgesic doses was also analyzed, compared using the Mann–Whitney test (intergroup).

Statistical analyses were performed using SPSS version 20.0, with a significance level of *p* < 0.05. Missing data were handled on an intention-to-treat basis, with an imputation of values from the second and third assessments.

## Results

**Fig. 2:**
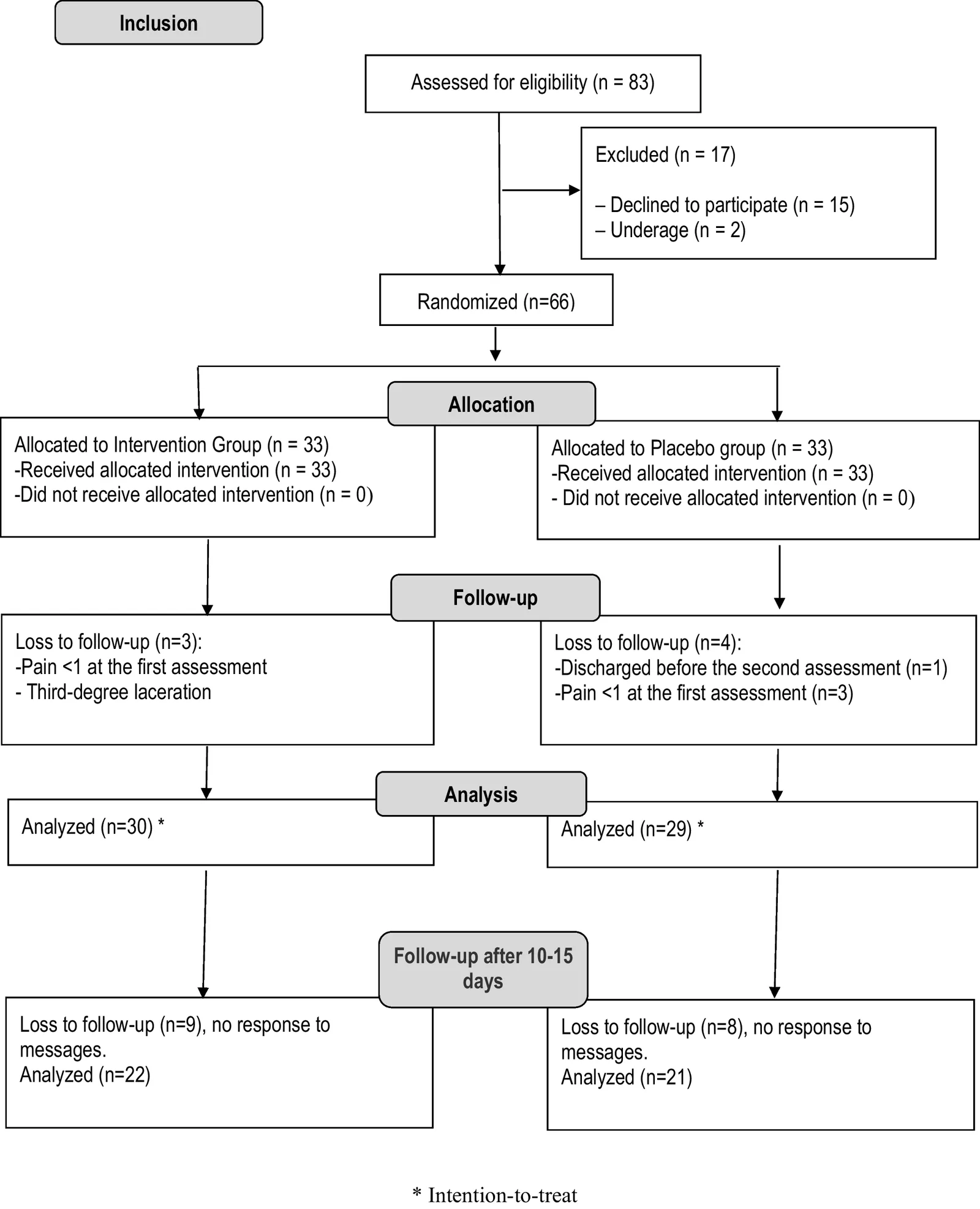
Flowchart adapted from Consolidated Standards of Reporting Trials (2025). Source: Prepared by the author

A total of 83 women were screened for eligibility; 2 were excluded because they were minors, and 15 because they refused to participate. After the randomization, 1 woman was excluded because she was discharged before the second assessment, 5 because they reported pain of less than 1 (NRS < 1) at the first assessment, and 1 because of a third-degree laceration. A total of 59 women were included in the analysis (30 in the intervention group and 29 in the placebo group). Ten women (3 from the intervention group and 7 from the placebo group) were discharged before the third assessment; therefore, the results were analyzed using the intention-to-treat approach (Fig. 2). The data collection took place between September 2024 and January 2025.

Table 1 presents the characteristics of the study population. The groups were homogeneous across sociodemographic and clinical factors perinatal, as well as deliveries conditions.

**Table 1:** Demographics and clinical characteristics of the study population

Variables	Intervention group (*n* = 30)	Placebo group (*n* = 29)	*p* value
Maternal age (years)	29.07 ± 1.25	27.79 ± 1.28	0.48*
Self-reported race/color			
Brown-skinned	4–13.3%	10–34.5%	0.12**
Black	2–6.7%	4–13.8%	
White	23–76.7%	15–51.7%	
Asian	1–3.3%	0–0%	
Comorbidities			
Yes	4–12.9%	3–9.7%	0.69**
No	27–87.1%	28–90.3%	
Parity			
Primiparous	19–63.3%	15–51.7%	0.37**
Multiparous	11–36.7%	14–48.3%	
Number of childbirths	0.53 ± 0.19	0.43 ± 0.11	0.49*
Cesarean sections	0.05 ± 0.05	0.03 ± 0.19	0.98*
Vaginal deliveries	0.26 ± 0.10	0.33 ± 0.11	0.32*
Number of miscarriages	0.26 ± 0.10	0.14 ± 0.78	0.85*
Gestational age at childbirth (weeks)	38.86 ± 0.59	37.92 ± 0.44	0.52*
Second stage of labor (min)	158.95 ± 27.81	188. 67 ± 32.03	0.31*
Episiotomy			
Yes	3–10.0%	2–6.9%	0.52**
No	27–90%	27–93.1%	
Laceration			
First-degree	14–46.7%	13–46.4%	0.58**
Second-degree	16–53.3%	15–53.6%	
Newborn weight (g)	2,870.82 ± 163.82	2,938.57 ± 110.45	0.52*

The characteristics of the study population were summarized based on demographic variables (age, skin color) and clinical variables (comorbidities and obstetric history), as well as delivery-related conditions (gestational age, newborn weight, duration of the expulsive phase, and presence of laceration)

*Mann–Whitney test

**Chi-squared test

Table 2 presents the means, standard deviations, and statistical analysis of pain variables in the intervention group and placebo group in the four assessments. There was no statistically significant difference in the intergroup analysis for any of the pain variables. Between the pre-intervention assessment and post-intervention 1 there was a significant reduction in perineal pain in both groups during rest and during movements. Between the pre-intervention assessment and post-intervention 2 the reduction remained significant for pain during rest and movements in both groups.

**Table 2:** Primary outcome: pain measured by numerical rating scale for pain, assess pre- and post-intervention and at follow-up

Variables	Intervention	Intervention group (*n* = 30)			Placebo group (*n* = 29)			
		NRS (0–10), mean ± SD	Comparators	Intragroup analysis (ITT), *p* value (95% CI)	NRS (0–10), mean ± SD	Comparators	Intragroup analysis (ITT), *p* value (95% CI)	Intergroup analysis (ITT), *p* value (95% CI)
Pain (at rest)	Pre-intervention	4.23 ± 2.84	Pre/post 1	0.00* (1.12–3.80)	3.45 ± 3.94	Pre/post 1	0.01* (0.25–3.55)	0.71 (−2.58–1.02)
	Post-intervention 1 (6–12 h)^a^	1.77 ± 2.30	Pre/post 2	0.00*(2.10–4.56)	1.55 ± 2.03	Pre/post 2	0.00* (1.07–4.19)	0.88 (−1.35–0.91)
	Post-intervention 2 (12–18 h) ^a^	0.90 ± 1.81	Post 1/post 2	0.27 (−0.20–1.94)	0.82 ± 1.47	Post 1/post 2	0.14 (−0.20–1.66)	0.86 (−0.94–0.78)
	Follow-up (7–15 days)^b, c^	1.05 ± 1.75	–	–	1.38 ± 0.50	–	–	0.67(−0.35–1.01)
Pain (during movements)	Pre-intervention	4.76 ± 2.84	Pre/post 1	0.02* (1.09–3.85)	4.30 ± 2.57	Pre/post 1	0.02* (0.27–3.03)	0.60 (−1.87–0.95)
	Post-intervention (6–12 h)^a^	2.29 ± 2.49	Pre/post 2	0.01*(0.97–3.73)	2.65 ± 2.67	Pre/post 2	0.00* (1.31–3.71)	0.58 (−0.99–1.71)
	Post-intervention (12–18 h)^a^	2.41 ± 2.53	Post 1/post 2	0.93 (−1.42–1.18)	1.79 ± 1.96	Post 1/post 2	0.04* (−0.37–2.09)	0.53 (−1.80–0.56)

The table presents the primary outcome, analyzing by ITT, during the pre-intervention (6–12 h postpartum), post-intervention 1 (6–12 h post first intervention), post-intervention 2 (12–18 h post-first intervention) and follow-up (7–15 days postpartum) in the intervention group and the placebo group

*CI* confidence interval, *ITT* intention to treat, *NRS* Numeric Rating Scale, *SD* standard deviation

In the rows: *p* value referring to intergroup analysis–Mann–Whitney test. Only the hygiene variable used the unpaired *t* test

In the columns: *p* value referring to intragroup analysis–Friedman test. Only the hygiene variable used repeated measures ANOVA and Bonferroni post hoc test

*p < 0.05.

^a^Hours post-first intervention

^b^Days postpartum

^c^Without in-person assessment

Table 3 presents the wound-healing variables in the intervention group and in the placebo group in the three assessments. There was no statistically significant difference in the intergroup analysis for any of the wound-healing variables. In the intragroup analysis, between the pre-intervention assessment and post-intervention 1, there was a significant reduction primarily in inflammatory signs (hyperemia, edema, and ecchymosis) and in the total healing score in both groups. Between the pre-intervention assessment and the post-intervention 2 assessment, the improvement remained significant for most variables, including hyperemia, edema, ecchymosis, and total score, in both groups. The secretion variable showed a significant reduction only in the intervention group. In the comparison between the post-intervention 1 assessment and the post-intervention 2 assessment, significant reductions were observed, particularly in the total healing score, in both groups.

**Table 3:** Secondary outcome: healing measured by redness, edema, ecchymosis, discharge, approximation (REEDA), assess pre- and post-intervention

Variables	Intervention	Intervention group (*n* = 30)			Placebo group (*n* = 29)			
		REEDA Scale, mean ± SD	Comparators	Intragroup analysis (ITT), *p* value (95% CI)	REEDA Scale, mean ± SD	Comparators	Intragroup analysis (ITT), *p* value (95% CI)	Intergroup analysis (ITT), *p* value (95% CI)
Hyperemia	Pre-intervention	2.63 ± 0.72	Pre/post 1	0.00* (0.70–1.50)	2.38 ± 0.98	Pre/post 1	0.00* (0.50–1.44)	0.38 (−0.70–0.20)
	Post-intervention 1 (6–12 h)^*b*^	1.53 ± 0.82	Pre/post 2	0.00* (1.52–2.20)	1.41 ± 0.78	Pre/post 2	0.00* (1.07–1.97)	0.53 (−0.54–0.30)
	Post-intervention 2 (12–18 h) ^b^	0.77 ± 0.57	Post 1/post 2	0.00* (0.40–1.12)	0.86 ± 0.71	Post 1/post 2	0.06 (0.16–0.94)	0.67 (−0.25–0.43)
Edema	Pre-intervention	2.33 ± 0.88	Pre/post 1	0.00* (0.42–1.30)	2.24 ± 0.95	Pre/post 1	0.00* (0.47–1.45)	0.75 (−0.51–0.33)
	Post-intervention 1 (6–12 h)^*b*^	1.47 ± 0.82	Pre/post 2	0.00* (1.07–1.93)	1.28 ± 0.92	Pre/post 2	0.00* (1.07–1.99)	0.43 (−0.64–0.26)
	Post-intervention 2 (12–18 h) ^b^	0.83 ± 0.79	Post 1/post 2	0.01* (0.22–1.06)	0.71 ± 0.81	Post 1/post 2	0.07 (0.11–1.03)	0.54 (−0.54–0.30)
Ecchymosis	Pre-intervention	1.97 ± 1.16	Pre/post 1	0.00* (0.36–1.44)	1.86 ± 1.16	Pre/post 1	0.00* (0.44–1.56)	0.72 (−0.71–0.49)
	Post-intervention 1 (6–12 h)^b^	1.07 ± 0.92	Pre/post 2	0.00* (1.02–1.96)	0.86 ± 0.95	Pre/post 2	0.00* (0.77–1.81)	0.39 (−0.70–0.28)
	Post-intervention 2 (12–18 h) ^b^	0.48 ± 0.57	Post 1/post 2	0.02* (0.19–0.99)	0.57 ± 0.79	Post 1/post 2	0.29 (−0.17–0.75)	0.98 (−0.27–0.45)
Secretion	Pre-intervention	1.30 ± 1.51	Pre/post 1	1 (−0.78–0.78)	1.07 ± 1.41	Pre/post 1	0.84 (−0.67–0.81)	0.52 (−0.99–0.53)
	Post-intervention 1 (6–12 h)^b^	1.30 ± 1.51	Pre/post 2	0.02* (−0.27–1.07)	1.00 ± 1.41	Pre/post 2	0.16 (−0.41–0.97)	0.42 (−1.06–0.46)
	Post-intervention 2 (12–18 h) ^b^	0.90 ± 1.06	Post 1/post 2	0.02* (−0.27–1.07)	0.79 ± 1.20	Post 1/post 2	0.23 (−0.48–0.90)	0.64 (−0.70–0.48)
Coaptation	Pre-intervention	0.77 ± 0.93	Pre/post 1	0.05 (−0.73–0.05)	0.72 ± 0.92	Pre/post 1	0.1 9(−0.10–0.72)	0.86 (−0.53–0.43)
	Post-intervention 1 (6–12 h)^b^	0.43 ± 0.50	Pre/post 2	0.05 (−0.05–0.73)	0.41 ± 0.63	Pre/post 2	0.05 (−0.04–0.76)	0.65 (−0.32–0.28)
	Post-intervention 2 (12–18 h) ^b^	0.43 ± 0.50	Post 1/post 2	1 (−0.26–0.26)	0.36 ± 0.56	Post 1/post 2	0.69 (−0.26–0.36)	0.46 (−0.35–0.21)
Total score	Pre-intervention	9.07 ± 4.67	Pre/post 1	0.00* (−5.64–1.12)	8.28 ± 4.68	Pre/post 1	0.00* (1.01–5.61)	0.48 (−3.23–1.65)
	Post-intervention 1 (6–12 h)^b^	5.69 ± 4.04	Pre/post 2	0.00* (3.72–7.82)	4.97 ± 4.06	Pre/post 2	0.00* (2.89–7.33)	0.49 (−2.83–1.39)
	Post-intervention 2 (12–18 h) ^b^	3.30 ± 3.09	Post 1/post 2	0.00* (0.53–4.25)	3.17 ± 3.74	Post 1/post 2	0.02* (−0.25–3.85)	0.64 (−1.92–1.66)

The table presents the second outcome, analyzing by ITT, during the pre-intervention (6–12 h postpartum), post intervention 1 (6–12 h post-first intervention) and post-intervention 2 (12–18 h post-first intervention) in the intervention group and the placebo group

*CI* confidence interval, *ITT* intention to treat, *REEDA* redness, edema, ecchymosis, discharge, and approximation, *SD* standard deviation

In rows: *p* value for intergroup analysis–Mann–Whitney test

In columns: *p* value for intragroup analysis–Friedman test

*p < 0.05

^a^Days postpartum

^b^Hours post-first intervention

Tables 4 and 5 present the frequencies and percentages of analgesic use in the intervention group and the placebo group across the assessments. In the intergroup analysis, a statistically significant difference was observed only for the use of simple analgesics in the post-intervention 1 assessment. In the intragroup analysis, the intervention group showed a significant reduction in the use of simple analgesics between the pre-intervention and the post-intervention 2 assessment, as well as between the post-intervention 1 and post-intervention 2 assessment. For NSAIDs, a significant difference was observed only in the intervention group between the pre-intervention and post-intervention 2 assessment. There were no significant differences in the use of opioid analgesics. There was a statistically significant difference in the intergroup analysis for the interval between the first and second doses.

**Table 4:** Additional outcome: medication use pre- and post-intervention

Variables	Groups	Use	Pre-intervention	Post-intervention 1 (6–12 h)^b^	Post-intervention 2 (12–18 h) ^b^	Intragroup analysis (*p* value)		
						Pre/post 1	Pre/post 2	Post 1/post 2
Simple analgesic	Intervention	Yes	19–63.3%	17–56.7%	8–26.7%	0.55	0.01*	0.01*
		No	9–30%	11–36.7%	19–63.3%			
	Placebo	Yes	12–1.40%	7–24.10%	9–31.00%	0.33	0.58	0.73
		No	14–8.30%	19–65.50%	17–58.30%			
Intergroup analysis, *p* value			0.11	0.01*	0.70			
Opioid analgesic	Intervention	Yes	4–13.3%	5–16.7%	4–13.3%	0.71	1.0	0.74
		No	24–80%	23–76.7%	23–76.7%			
	Placebo	Yes	7–24.10%	3–10.30%	3–10.30%	0.22	0.22	1.0
		No	19–5.50%	23–79.30%	23–79.30%			
Intergroup analysis, *p* value			0.25	0.51	0.73			
NSAID	Intervention	Yes	21–7%	17–56.7%	14–46.7%	0.29	0.04*	0.49
		No	7–23.3%	11–36.7%	13–43.3%			
	Placebo	Yes	17–58.6%	10–34.5%	11–37.9%	0.12	0.07	1.0
		No	9–31.0%	16–55.2%	15–51.7%			
			0.44	0.10	0.49			

The table presents additional outcomes. NSAIDs, opioid analgesics, and simple analgesics: during the pre-intervention, post-intervention 1 (6–12 h post-first intervention) and post-intervention 2 (12–18 h post-first intervention) in the intervention group and the placebo group

*CI* confidence interval, *NSAIDs* nonsteroidal anti-inflammatory drugs

In rows: *p* value for intergroup analysis–Chi-squared test

In columns: *p* value for intragroup analysis–Cochran’s Q test

*p < 0.05

^a^Days postpartum

^b^Hours post-first intervention

**Table 5:** Additional outcome: Medication doses

		Between dose 1 and dose 2	Between dose 1 and dose 3	Between dose 2 and dose 3
Simple analgesic-dose intervals (h)	Intervention	4.11 ± 4.92	5.85 ± 11.09	2.77 ± 6.20
	Placebo	0.96 ± 2.84	3.92 ± 9.26	0.28 ± 1.40
Intergroup analysis, *p* value (95% CI)		0.01* (5.25–1.05)	0.46 (−7.27–3.41)	0.05 (0.16–4.82)

The table presents additional outcomes

*CI* confidence interval

Simple analgesic-dose intervals: in rows *p* value for intergroup analysis–Mann–Whitney test

*p < 0.05

Concerning the women’s satisfaction with the intervention, only the intergroup analysis was performed, with a mean and standard deviation of 1.19 ± 0.40 for the intervention group and 1.10 ± 0.57 for the placebo group, with no statistical difference (*p* = 0.38). In self-perception of perineal laceration healing, the mean and standard deviation were 1.24 ± 0.54 in the intervention group and 1.05 ± 0.50 in the placebo group (*p* = 0.15) respectively.

No discomfort or side effects were reported by the participating women.

## Discussion

The study found no significant difference in intergroup analysis in terms of pain reduction and healing following the application of a PBM protocol (660 nm; 4 J) in women with postpartum lacerations or episiotomies.

The results of the present study are consistent with previous research. Although most studies have not identified a significant reduction in pain intensity [17–20], Boniatti et al. [21] found pain reduction when comparing PBM with cryotherapy.

A systematic review with a meta-analysis indicated that, to date, there has been low-quality evidence, with heterogeneity in protocols and inconclusive results regarding pain reduction and healing after PBM [13]. Studies conducted so far have predominantly used low doses of PBM and short-duration protocols, not exceeding 1 week of intervention [13].

Pain assessment methods show a considerable heterogeneity across studies [17–21]. Although the NRS is the most widely used instrument, its use alone may represent a limitation owing to its subjectivity [18–20]. The Short-Form McGill Pain Questionnaire was used in conjunction with the NRS in two studies, potentially providing a more comprehensive assessment [17, 21]. Direct methods of pain assessment, such as algometry [14], could be incorporated into the measurement, as well as mixed methods that include qualitative variables.

The benefits of PBM during movements following perineal lacerations may become more evident over the long term when assessed through specific functional evaluations, such as the sit-to-stand test and the Timed Up and Go test.

To our knowledge, no study to date has demonstrated satisfactory results regarding the healing process [13, 17–21], possibly because of the short follow-up period. In the present study, healing was assessed within the first 24 h after injury, a period characterized by acute inflammatory signs. The inflammatory phase begins immediately after the injury and can last up to approximately 4 days, whereas the proliferative phase becomes more evident at between 5 and 7 days [22]. Thus, the effects of PBM on tissue repair may be more noticeable in long-term evaluations, when the proliferative phase predominates. However, in the acute period, PBM may contribute to the control of inflammatory signs, such as pain, edema, and hyperemia [14].

The REEDA scale was used in all studies investigating tissue repair following a perineal laceration [17–21]. The scale can be administered within the first 24 h, as it includes items related to the inflammatory phase, and up to 10 days after the injury [8]. Two studies that conducted in-person follow-up between 7 and 20 days postpartum using the REEDA scale observed no differences in intergroup analyses and reported significant loss to follow-up, which may have compromised the results [18, 19]. In this study, we opted for an assessment via text messages owing to the challenges of the postpartum period, such as adapting to a new routine and maternal exhaustion, which can reduce adherence to the in-person follow-up. However, objective methods, such as the REEDA scale, should be preferred.

Statistical differences were observed in the intragroup analyses for both groups for the variables of pain and wound healing. In the additional analysis, the frequency of simple analgesic use in the intervention group decreased significantly over time. The interval between doses of simple analgesics increased significantly in the intervention group compared with the placebo group. There was no difference in the intergroup analysis regarding women’s satisfaction with the intervention or self-perception of perineal laceration healing.

The reduction in pain intensity in both groups can be explained by the women’s natural recovery during the postpartum period [13]. At the same time, social, physical, and emotional adaptations may increase susceptibility to the placebo effect, with psychological and potential therapeutic impacts [23, 24]. This may have contributed to the similarity between the results of the placebo and intervention groups, as women in the placebo group also felt cared for and listened to [23, 24]. This may also explain the lack of difference in the intergroup analysis regarding women’s satisfaction with the intervention.

Additionally, medication consumption was analyzed in this study. There was no intergroup difference for NSAIDs and opioid analgesics, which are part of the standard maternity ward prescription for perineal lacerations. However, the intervention group showed a significant difference in the number of doses and the interval between them for simple analgesics, the administration of which depends on the woman’s need. These results could have clinical relevance, considering that most maternal medications are transferred to the infant via breastfeeding [25].

From an institutional perspective, reducing medication use and adopting PBM may contribute to cost containment, greater service efficiency, and a potential reduction in the length of stay [26, 27]. However, the findings should be interpreted with caution owing to the risk of bias, and these results could be influenced by the natural progression of the postpartum recovery process or by multiple statistical tests. Cost-effectiveness studies are necessary, as the clinical benefits of PBM are not yet fully understood, and its implementation involves expenses for equipment and consumables [28].

The results of the present study demonstrate imprecise clinical implications, and limitations must be acknowledged. The absence of more comprehensive instruments may have limited the effectiveness of pain assessment, in addition to the routine use of medications in the hospital setting. The absence of an in-person assessment using the REEDA scale within 7–10 days after the injury compromises evaluation of the effect of PBM on the healing process. Furthermore, the long-term effects of PBM on perineal pain during movements were not evaluated. In additional analysis, the relevance of the reduction in simple analgesic consumption is unclear owing to the high risk of bias.

For future research, it is recommended to use direct and comprehensive methods for pain assessment, besides incorporating qualitative evaluations. The objective and in-person assessment of the long-term healing process should be considered (7–10 days). Intervention protocols lasting longer than 1 week need to be investigated. The use of analgesics requires more effective methods of assessment and control to reduce the risk of bias. Conducting functional tests during evaluations, as well as cost-effectiveness studies of the intervention, could answer clinical questions that have not yet been addressed on this topic. Women’s satisfaction with the intervention should be further explored.

In conclusion, the use of PBM did not demonstrate a significant reduction in perineal pain at rest and during the functional movements compared with the placebo group within 24 h after perineal lacerations, nor did it show a significant acceleration in the healing process. A statistically significant decrease in analgesic use was observed in the intervention group, which could be due to the natural progression of the postpartum recovery process. Further studies may shed light on the effects of PBM on perineal lacerations and episiotomies.

## Data Availability

The datasets generated and/or analyzed during the current study are available from the corresponding author upon reasonable request.
